# Experience Matters: Females Use Smell to Select Experienced Males for Paternal Care

**DOI:** 10.1371/journal.pone.0007672

**Published:** 2009-11-04

**Authors:** Nichola Fletcher, Ellen J. Storey, Magnus Johnson, Donald J. Reish, Jörg D. Hardege

**Affiliations:** 1 Department of Biological Sciences, University of Hull, Hull, United Kingdom; 2 Centre for Environmental and Marine Research, University of Hull, Scarborough, United Kingdom; 3 Department of Biological Sciences, California State University, Long Beach, California, United States of America; University of Exeter, United Kingdom

## Abstract

Mate choice and mating preferences often rely on the information content of signals exchanged between potential partners. In species where a female's reproduction is the terminal event in life it is to be expected that females choose high quality males and assess males using some honest indicator of male quality. The Nereidid polychaete, *Neanthes acuminata*, exhibits monogamous pairing and the release of eggs by females terminates her life and larval success relies entirely on a male's ability to provide paternal care. As such females should have developed reliable, condition-dependent criteria to choose mates to guarantee survival and care for offspring. We show that females actively chose males experienced in fatherhood over others. In the absence of experienced males dominance, as evident from male-male fights, is utilized for mate selection. The preference for experienced males is not affected by previous social interactions between the individuals. We show that the choice of the partner is based on chemical signals demonstrating a ‘scent of experience’ to females providing evidence for the role of chemical signals in sexual selection for paternal care adding to our understanding of the mechanisms regulating condition-dependent mate choice.

## Introduction

The operation of sexual selection by male-male competition and female choice has been the subject of extensive empirical, as well as theoretical analysis [1], [2] and few today would doubt its impact on the evolution of phenotypes [3], on the dynamics of populations [4] and on reproductive isolation [5]. However, some areas of this bourgeoning field are yet to be fully explored and understood. Fundamental questions, only partly resolved include the criteria of female choice [6], [7]. Females in many species show preference for dominant males; this can either be assessed by male – male competition, the winner proving to be of superior quality to that of its opponents, or traits that reflect its status [1]. It is typically also difficult to disentangle components of sexual selection, i.e. direct sexual selection on males by female choice versus indirect sexual selection, where intrasexual selection or competition between males acts independently [8].

In incidents of direct mate choice, females might be choosing for direct benefits such as nuptial gifts from males [4] or protection from harassment by other males [6]. Alternatively, the object of female choice might be traits in males that signal indirect benefits, i.e. beneficial traits transmitted to the female's progeny [9]. The amount of parental care provided is known to exert strong selection on mating preferences. When males exclusively care for the young, and male quality in parental care is genetically inherited, females should prefer good fathers. This could provide direct benefits in the form of higher survival of the clutch and indirect benefits by generating sons with good parenting abilities, or both [9], [10].

Parental care skills might also be acquired through experience and females would therefore be expected to choose experienced males [11]. There is some evidence for this in some teleosts [12] and birds [13] in which females lay their eggs in the nests of males already caring for eggs. Such badges of parental ability might be strongly selected when female opportunities for reproduction are limited and paternal care is indispensable to ensure offspring survival [14]. Extreme cases of such conditions include semelparous species where a lifetime's reproductive success depends on a single act of mate choice [15].

Already proposed by Darwin [16] as a key mechanism by which sexual selection might be promoted, the role of chemical signals in mate choice, when compared to other communication channels, is still poorly understood today [17]. Recent studies challenge the traditional view that chemical signals are simply used to facilitate species or mate recognition and merely induce stereotyped behavior leading to mating synchronization. Johansson and Jones [17] pointed out that experimental evidence is largely suggestive of the ability by the receiver of a chemical signal to detect its quality and quantity and that large variance exists in both these components between populations, between individuals and between single spawnings. As such, chemical signals can potentially convey a large amount of information but direct experimental evidence for mate quality assessment and mate choice based on chemical signals has lagged behind [17] with only few exceptions such as the role cuticular hydrocarbons play in *Drosophila birchii* and *D. serrata* [18].

The use of chemical signals in aggressive and sexual contexts is widespread in Nereidid polychaetes, this group representing among the very few examples of invertebrate taxa for which chemical communication has been characterized at the molecular level [19]. In *Neanthes acuminata*, fighting behavior forms the basis for dominance hierarchies within populations [20], and is regulated by complex chemical signalling [21], [22] that have even been suggested to be responsible for the evolution of reproductive isolation between the East and West Coast North-American populations of this species [23]. Although this theory and the interpretation of the empirical evidence remain controversial [24], male-male competition mediated by pheromonal exchange in *N. acuminata* is intense [21], [24].

*N. acuminata* is a female semelparous polychaete with exclusive female mate choice [21], pair formation, and exclusive male parental care following the female's death during reproduction [20], [25], without which no eggs will survive to the larval stages [26], [27]. This makes this species an ideal model system to address fundamental questions regarding the interactions between sexual selection and parental care and the role of chemical communication. Since a female's lifetime reproductive success depends on a single reproductive event, females should exert prudent choice and allocate their limited reproductive resources to males that can provide reliable evidence of successful paternal care during offspring development. *N. acuminata* males mate and care for young up to 7 times during their lifetime and populations of *N. acuminata* typically include sexually active, but inexperienced males and those who have fathered offspring before, i.e. experienced males. It is therefore possible but not yet experimentally proven that males gain paternal ability skills over their lifespan with increasing fathering experience.

Sexual selection is also often driven by male competition and subsequent female mate choice, and is thought to be the driving force behind many speciation events [1]. Females in many species including mammals [1], [5], insects [7], birds [14], and fish [3] show preference for dominant males. This is often assessed by male – male competition with the winner proving to be of superior quality, or possessing traits that reflect its status [1], [8]. It is therefore to be expected that females choose high quality males and assess males using honest indicators of male quality, this also being described as condition dependent mate choice. To examine the parameters that females utilize to choose a male partner, we initially tested whether reproductively active males engage in fighting behaviour when exposed to each other and whether this results in winner/loser dominance relationships. For female driven mate choice, we tested the hypothesis that semelparous female worms choose a male upon the perceived capability of the male to nurture her offspring following her death after the spawning event. We predict that a female's choice for experience is independent of a male's dominance status i.e. that a paternally experienced male will be preferred as a partner even if he has lost male-male fights. We also tested the hypothesis that females will recognize male paternal experience chemically to choose experienced fathers. Finally, we also predict that when exposed to two inexperienced males who have no paternal experience the female should prefer a dominant male over a subdominant male as partner.

## Results

**Figure 1:**
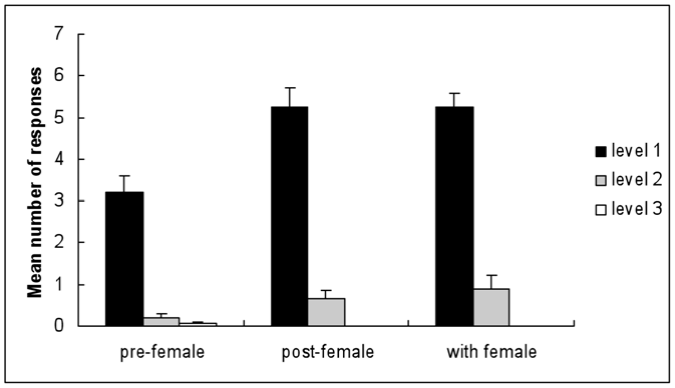
Male-male aggression between inexperienced males. Mean (± S.E.) number of aggressive responses in encounters between two inexperienced *N. acuminata* males (N = 40). Males were allowed to interact (fighting) prior to having female contact (pre-female); after pairing with a female (post-female); and with a female present (with female). Aggression levels increased significantly when the two males were in contact with a sexually mature female and stayed high after the female contact indicating that the fighting intensity is resource-driven. Three different aggression levels were recorded; (1) palps flared and avoidance (black bar), (2) fighting position with palps flared and jaws visible (grey bar) and (3) attacking behavior, biting and grasping (white bar).

Male-male aggression encounters between inexperienced males were observed to examine the effects of female presence. There were significantly different levels of aggression observed between the frequencies recorded (Friedman test  = 104.9, df = 2, p = 0.0001) with the majority of males found to actively avoid each other with their palps flared (level 1 aggression, Fig. 1). Males showed different frequencies of level 1 aggression dependent on exposure to a receptive female (Friedman test  = 15.972, df = 2, p = 0.0003). Males were more aggressive after pairing with a female than prior to contact (Wilcoxon signed-ranks test, *Z* = −2.99, N = 20, p = 0.0031, Bonferroni corrected significant to 0.005 level) and similarly showed increased aggression in the presence of a female (Wilcoxon signed-ranks test, *Z* = −3.552, N = 20, p = 0.0004). However, males did not fight with females and did not force females into pair formation. No difference in frequencies of aggression were seen (Fig. 1) between males allowed to previously pair with a female or with a female present (Wilcoxon signed-ranks test, *Z* = −0.07, N = 20, p = 0.9430). No significant difference was observed between all 3 conditions at level 2 (Friedman test  = 4.65, df = 2, p = 0.098) or level 3 aggression (Friedman test  = 2, df = 2, p = 0.368).

**Figure 2:**
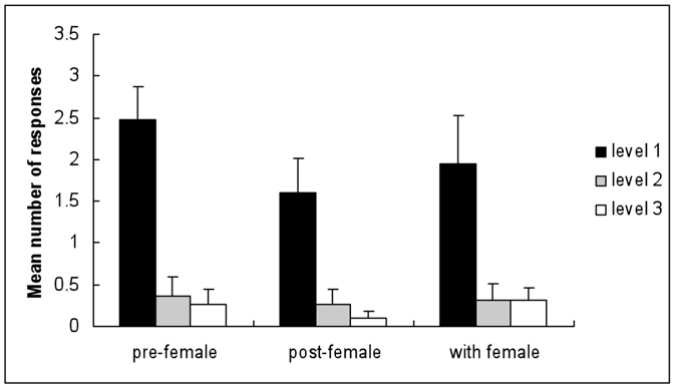
Male-male aggression between males experienced in fatherhood and inexperienced males. Mean (± S.E.) number of aggressive responses in encounters between experienced (having successfully fathered offspring, N = 19) when in contact with inexperienced *N. acuminata* males (N = 19). Males were allowed to interact (fighting) prior to female contact (pre-female), after pairing with a female (post-female), and with a female present (with female). Three different aggression levels were recorded; (1) palps flared and avoidance (black bar), (2) fighting position with palps flared and jaws visible (grey bar) and (3) attacking behavior, biting and grasping (white bar).

We also examined aggressive encounters between inexperienced males and experienced males. Individuals showed significantly higher levels of avoidance behaviour (level 1; Fig. 2) than any other aggression level (Friedman test  = 24.6, df = 2, p = 0.0001). Individuals showed no differences in level 2 or level 3 aggression prior, post female contact or with a female present (Friedman test  = 1.9, 0.4, 0.6 respectively, df = 2, p = 0.387, 0.819, 0.741).

**Figure 3:**
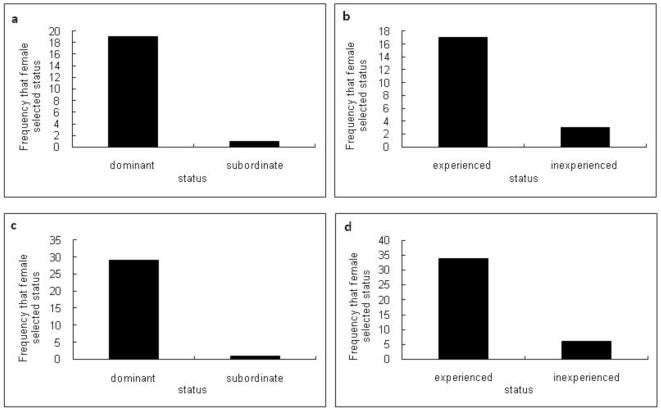
Female mate choice for experience and dominance male traits. The frequency that in mate choice experiments the female *Neanthes acuminata* chose specific male traits. Figure 3a & 3c shows the choice of dominant males over subordinate males, and in Figure 3b & 3d chose males that were experienced in ‘fatherhood’ over an inexperienced male. Two sets of experiments were undertaken using a choice chamber: bioassays 3a & 3b were performed with no social interaction involved prior to selection. Figure 3c & 3d are the results of the study performed with social interactions allowed between both males and the female prior to selection. Regardless of having pre-experiment interactions or not females significantly preferred dominant males over subordinates and experienced over inexperienced indicating that condition-dependent female choice exists in this species.

The effect of the dominant – subordinate relationships established in male-male encounters in step 1 of our study was examined in subsequent female mate choice experiments. We used non-social mating trials without prior contact between the potential partners to avoid memory effects. When exposed to two inexperienced males, the females chose dominant males over subordinate males, with 95% choosing the dominant male (Fig. 3a) without having any previous social interaction (χ^2^/_1_ = 16.25, p = 0.0003). Females also showed preference for experienced males (Fig. 3b) over inexperienced (χ^2^/_1_ = 9.85, p = 0.007), with 85% of females selecting and forming a pair bond with a male with previous experience of fatherhood.

When social interactions were allowed between both males and separate females, the female added in step 3 of our study initially remained undecided having physical contact with both males before pair bonding with one male. When comparing dominant (winner) males with subordinate (Fig. 3c), females chose the more dominant male (χ^2^/_1_ = 26.16, p = 0.0001). Similarly (Fig. 3d) the female chose the more experienced male (χ^2^/_1_ = 19.6, p = 0.0001) over inexperienced males.

Experienced males were not always the winner in male- male aggressive encounters, e.g. in 20 encounters without social interactions, experienced males dominated in 13 and inexperienced males in 7 fights, 12∶8 in fights with social interactions. When analysing female preference data for 40 male-male encounters where a clear dominance status could be assigned for in the fights, the females chose 25 (62.5% of total males chosen; 25/25 of E/W males) males that were dominant and experienced (E/W). This is significantly higher than the other encounters (χ^2^/_1_ = 19.22, p<0.0001) where 10 (25% of total males chosen; 10/15 of E/L males) of the males chosen were subordinate (loser) but experienced males (E/L). Whilst 35 of the 40 males chosen were experienced males, only 5 (12.5% of total males chosen; 5/15 of IE/W males) were inexperienced males and dominant (winner) individuals (IE/W). None of the males that were chosen were inexperienced and at the same time subordinate (loser; IE/L; 0% of total males chosen; 0/25 of IE/W males) showing that experience is the highest priority for females when choosing a male.

**Figure 4:**
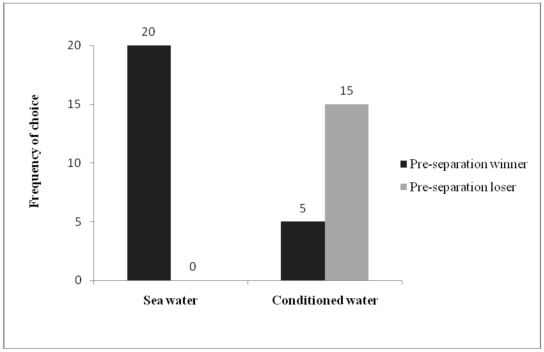
The role of scent of experience in mate choice. The frequency of female's changing mate choice when previously rejected males were masked with ‘scent of experience’. Following separation males were kept in seawater as a control (N = 20), and in ‘conditioned water’ collected from males nursing eggs (N = 20) and presented to the females for mate choice. The smell of males nursing young prompted 75% of the females to accept the pseudo-experienced male showing that odour compounds are responsible for female mate choice for experienced males.

Masking inferior males with the ‘smell of experience’ should potentially transform these males into preferred partners if chemical signals are involved. We conducted further female mate choice experiments to confirm changes in mate choice of females due to scent masking. After a period of separation in seawater, females were given a subsequent choice between two inexperienced males, of which one was previously rejected (loser). The female chose the same male (winner) as was selected pre-treatment (100% of cases). However, masking of the loser of pre-treatment female choice with ‘conditioned water’ from males that were nursing young had a significant impact upon the subsequent choice of the female. In 75% of cases (χ^2^/_1_ = 20.91, p = 0.0001), females altered their choice from the pre-treatment winner to the pre-treatment loser (Fig. 4).

## Discussion

Our data show that when *N. acuminata* males encounter each other aggression and fighting occurs. There were significant differences in the aggression levels prior, post and with female contact between dominant and subordinate males (Fig. 1). In *N. acuminata*, individuals of the same sex engage in aggressive encounters for example over food or to fight off an intruder of either sex during parental care [26]. Aggressive encounters resulted in many instances, but not always, in dominant and subordinate (winner/loser) males [26]. Male dominance hierarchies are widespread and there is extensive literature to support the appearance of dominant-subordinate relationships [2]. Male aggression in *N. acuminata* is significantly related to intra-sexual competition for the possession of the female and is as such resource driven (Fig. 1). Nevertheless, in *N. acuminata* this is an indirect process of increasing a male's chances of mating since this species has exclusive female mate choice [21], and males do not fight with females and do not force pair formation [20], [21]. Resource driven fighting may be associated with several aspects; one being the sex ratio, which in natural populations is 50∶50 [26]. A skewed Operational Sex Ratio (OSR) can lead to increased competition among members of the more abundant sex. Changes in OSR can occur over the breeding season and have been described in female competition in the sand goby *Pomatoschistus minutus* [28]. A biased sex ratio can occur if one sex solely cares for offspring over the breeding season [29] and this would be expected for male *N. acuminata* parental care since the females die after reproduction. As the iteroparous males can fertilize eggs faster than the semelparous females produce them, females could be considered to be a scarce resource and subsequently be in demand, increasing the pressure on males to advertise quality.

Figure 2 shows that aggression levels in encounters between inexperienced and experienced males. Fighting intensity between the males did not significantly increase upon female contact, potentially indicating that a social hierarchy has been established where inexperienced males may not challenge experienced males for female partners. Male aggressive behaviour could have no influence on female mate choice in *N. acuminata*. This seems unlikely since female choice has been observed when females have a greater investment in reproduction and males have a higher potential reproductive output [30]. Alternatively, females use signals other than the aggressive visual displays to identify the quality of a male. This is evident through the lack of a significant difference in male-male aggression in the presence of a female when placed together after the initial male-male fight compared to the initial encounter pre-female exposure (Fig. 2). Females are expected to be more discriminating and select mates based on traits that can increase female fitness. Male traits can signal that he is in good physical condition [30], can often be a sign of good genetic quality [31] and may indicate superior fighting ability, the quality of male parental care [32] and provision of breeding resources.

In our study, where we used those aggressive encounters between two inexperienced males that elicited a dominant/subordinate relationship the female choose the dominant male (Fig. 3a, 3c). Females select for dominance in many species including mammals, fish, insects, birds and invertebrates [10]. Often dominant males are larger in size and this may indicate that they are superior at fighting for access to mates or that they are better able to provide protection against predators [33]. Although male-to-male contests and the formation of dominance hierarchies are widespread, females do not always select for dominance [10]. For example, in the field cricket, *Teleogryllus commodus*, females do not prefer males with high fighting ability [34] and in cockroach *Nauphoeta cinerea* dominance and female choice are in opposition [35]. In species with male parental care, the ability to raise offspring can be the deciding factor in female mate choice if hatching success is heavily reliant on male care [36]. Dominance may not provide a good indication of parental care ability and dominant males do not always make better fathers [36]. Females choose males based on beneficial factors that provide benefits to the individual [7] but few studies exist where the criteria for choosing a male can be directly linked to a female's reproductive success [33]. We tested the hypothesis that female *N. acuminata* exhibit mate choice based on the good parent model of sexual selection, where males display the desired trait of care and rearing of a brood. *N. acuminata* chose experienced males over inexperienced (Fig. 3b & d) irrespective of witnessing interactions between the males. In this species, experience does not necessarily always result in dominance and in winning male-male fights. The choice of experienced fathers could be associated with the detection of higher quality in the male [3] and therefore the direct and indirect benefits this can offer the female. Experienced males have been shown to be preferred by several species of fish including sand gobies and sticklebacks [10], with these individuals benefiting through higher hatching success rates.

Sole male care is considered generally rare [37] but is seen in a number of fish species [36]. Investment in parental care should only occur if it does not negatively affect future reproductive success of the parent [38], i.e. due to the resource requirements by the offspring and reduced time to forage. This can lower future reproductive success and delay their return to the pool of potential mates [39]. In *N. acuminata* the iteroparous male has a trade-off between such disadvantages and gaining experience in fatherhood that results in preferential selection by future female partners, fitting the enhanced fecundity hypothesis [37]. Whether such males, especially inexperienced males, potentially exhibit a significant degree of paternal selfishness in guarding unrelated eggs, as postulated by Tallamy [37] as a key criteria for the existence of sexual selection in male paternal care, remains to be examined in future studies.

The semelparous female potentially optimizes the likelihood of reproductive success by mating with proven fathers. [31]. Experienced males potentially perform better in parental care and could add benefits to offspring as seen in flagfish, *Jordanella floridae* [40]. Tallamy [37] postulated that in all cases where paternal care occurs and females exhibit sexual selection, these females are iteroparous as otherwise they would not directly benefit from males willing to care. Our study shows that this is not the case in annelids such as *N. acuminata*. As male promiscuity in this species is constrained by the low number of females, selection should favor those males that invest postzygotic towards existing offspring over mate seeking [40]. That females chose experienced males demonstrates that the increased access to future female partners allows such traits to become preferred, even in species with semelparous females, fitting with the ‘enhanced fecundity’ hypothesis of trade-off between guarding and subsequent reproduction [41].

The decision to choose a male could be influenced by previous or experimental contacts between the individuals. Female choice for experienced males was independent of prior social interactions and observed aggressive behavior between the males (Figs. 3b & d). Most females chose the experienced males, regardless of whether they were dominant or subordinate in fights (Fig. 3d). In the absence of experienced males, females chose the dominant male of the two, again regardless of social interaction (Fig. 3a & 3c).

Chemical signalling is widespread in Nereidid polychaetes [19] and chemical signals are involved in mate recognition and in pre-mating isolation between different populations of *N. acuminata* [22]. Figure 4 demonstrates that *N. acuminata* also uses chemical cues for male status recognition, in this case experience in paternal care. Masking inexperienced males that were initially rejected by females (loser) with the scent of a male looking after young, led to 75% of the females changing their choice to now prefer the pseudo-experienced male loser over an inexperienced winner (Fig. 4). Whether this scent of experience is linked with a hormonal change during paternity in the male for example, similar to the increase in prolactin levels seen in fish, birds, and mammals [42], [43], or directly through taking on the scent of recent young during masking will require further investigation. Since males that are caring for young are known to aggressively attack any other worm that approaches their living tube, presumably to defend the young [26], the care for young can be seen as an investment into increasing their potential for future female mate choice. This makes it unlikely that the bioactive odor stems directly from the eggs/young, but instead signals the paternal quality of a male, this being similar to the adaptive female choice in lizards [44].

It is unclear whether experienced males are actually performing paternal care with a higher success rate than inexperienced males making it worthwhile for the female to prioritize for experienced males. In guppies, males prefer virgin females over recently mated females because of their higher reproductive value [45]. Our study provides evidence for chemical signal mediated female choice in a simple reproductive system. Semelparous females exhibit an active preference for traits displayed by an iteroparous male. These potentially maximize the female's reproductive success that depends entirely upon the iteroparous males. As such, *N. acuminata* could be an ideal system to study the mechanisms of paternal care and the role of chemical signals in sexual selection and condition-dependent mate choice in marine invertebrates, a field that despite recent progress is still in its infancy [17], [30], [35], [37].

## Materials and Methods

### Ethics Statement

All animal work using polychaete worms presented here has been conducted according to relevant national and international guidelines to ensure ethical appropriateness.

Most specimens used in this study originated from 6 worms from a population collected in 1964 from Los Angeles Harbor by one of us (Reish) and has now undergone over 200 generations in the laboratory. The worms used for the masking experiments however, were collected from Newport Beach by JDH in 2006 from an original sample of over 400 individuals and have therefore undergone only a few generations in the laboratory. Individuals were shipped by overnight air express to Hull University and maintained in aquaria tanks (76×30×37 cm), with aerated seawater (salinity 35–38‰), with the temperature kept at approximately 18°C. Worms were fed 3 times a week on a low protein rabbit food diet with partial water changes occurring fortnightly. To ensure the exact reproductive state of a male, juveniles were separated pre-maturity from the population and allowed to mature (evident by the male's sperm resulting in a whitening of the body [27]); any that developed into females were removed immediately. Upon maturity, 50% of the males were placed with females and allowed to breed. Those males that produced viable offspring and undertook parental care were considered ‘experienced in fatherhood’, and those not exposed to females were ‘inexperienced’. These groups were kept in isolated dishes.

### Male-Male Aggression Levels (Experimental Series 1)

**Figure 5:**
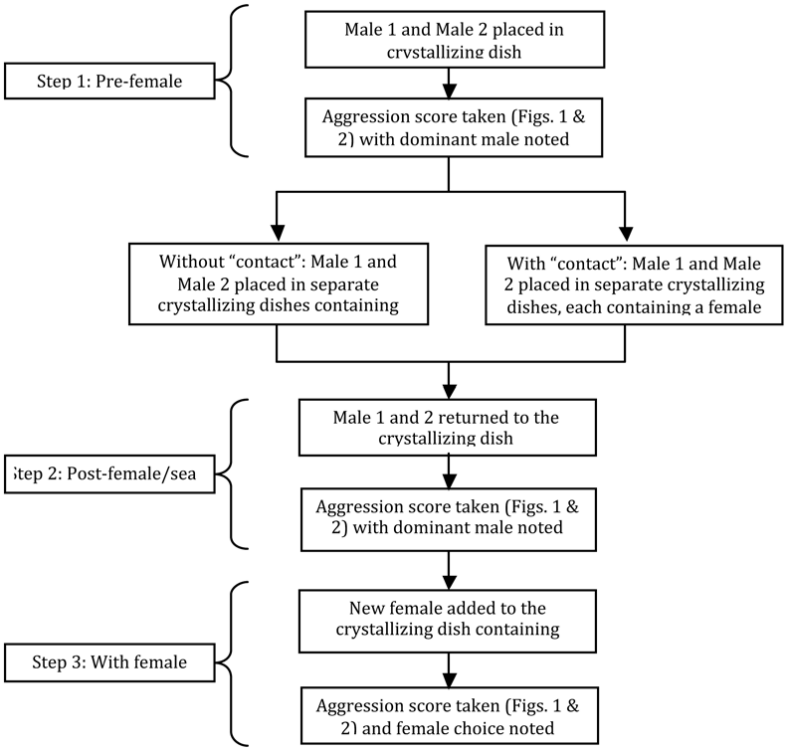
Flowchart showing the three steps of the experimental protocol and their links to the results (Figures) of the study.

Fighting and aggression is a common behavior in marine invertebrates and has been described in *N. acuminata* [20] mainly in male – male interactions [21]. We therefore initially investigated whether aggressive encounters between male worms led to dominance hierarchies pre-female exposure. For this we used either two inexperienced males (Fig. 1) or an inexperienced and an experienced male (Fig. 2), thus providing us also with data upon the question whether experienced males are more likely to win fights (see Figure 5). Aggression levels were monitored in accordance with the numerical scoring system slightly modified from Reish and Alosi [20] to measure the different intensities of fighting (Table 1).

**Table 1:** Scoring system for monitoring aggression levels in *Neanthes acuminata*.

Aggression level	Behavior
0	No fighting or avoidance behavior
1	Avoids contact with the opposition, palpi flared
2	Fighting position assumed with palpi flared and
	jaws visible, followed by immediate avoidance
3	Severe aggression and obvious attacks at the other
	specimen biting and grasping, obvious avoidance
	subsequent to these attacks

For the determination of male-male aggression, two males were placed in a crystallizing dish (80 mm in diameter) and aggression recorded using pin-point focal observations every 30 seconds over a period of five minutes. The worms were subsequently separated and placed individually with a female to test their reproductive status (ability to pair). Only those males that paired successfully with a female were used in the further studies. In the second phase of this experiment, successful males (N = 40 inexperienced males) were then subjected to the aggression bioassay to assess any changes in aggression levels post exposure to pairing with a female. In the final phase, a female was added to the arena and males were monitored for aggression levels in the presence of a female. This allowed for analysis of changes in aggression levels in different situations (motivation) and also the dominance status of the males.

Dominant animals (winners) were identified as those which displayed higher aggression levels than subordinates (losers), which retreated when in direct contact during a fight. This three-step bioassay was also undertaken with one male experienced in fatherhood (N = 19) versus an inexperienced male (N = 19), to determine aggression levels and dominance status (winner/loser) of individuals (see Figure 5).

### Female Mate Choice Experiments (Experimental Series 2)

Female mate choice bioassays were conducted to address the questions of whether females chose dominant (winner) males over subordinate (loser) males, and also if females use a male's experience as a father as a criteria for selection.

### Exposure of Female Worms to Males without Prior Social Contacts

Size-matched inexperienced males (one dominant; N = 20, one subordinate; N = 20), whose dominance status had previously been established as described above, were placed separately into each arm of a choice chamber designed using a partially (60 mm) divided crystallizing dish (80 mm in diameter) and allowed to acclimatize. In this species, individual worms often lay along the edge of the dish or into the corners of the partition with minimal movement [26] and therefore the worms did not need to be restricted during acclimatization. A reproductively active, mature female (noted by the presence of yellowing established ova in the coelom) was placed at the opposite end of the dish and left to choose between the two males, which were allowed to mix freely in the dish (recognized through individual colorization and pigmentation patterns). Following the approach of the female towards the males, female mate choice was observed when a female formed a pair bond with a male. Male-male aggression levels were also scored allowing us to distinguish between avoidance and fighting of the males (Table 1). This bioassay was repeated (N = 20) between males experienced in fatherhood and inexperienced males to see if females showed any preferences.

### Exposure of Female Worms to Males that Were Allowed Social Contacts

Additionally, we investigated whether a females' decision to choose a male was influenced through experimental social contacts between the individuals prior to the aggression level experiments. Initially, both males were allowed to interact and aggression levels were scored. Afterwards both males were separated and placed in crystallizing dishes, each containing a female (Fig. 5). After the pre-exposure to the female the males were placed back together and the aggression levels recorded again to examine whether the exposure to a female had affected their aggression potential. We then added a female to the males and allowed these females to witness aggression between the males (defined as ‘social contact’ throughout the manuscript) in order to determine if dominance or experience were preferred criteria for the female mate choice and whether the pre-exposure to the males did affect her choice.

Females (N = 30) were exposed to two inexperienced males in a crystallising dish arena (80 mm in diameter), allowing free mixing between both males and the female. The choice made by the female for a male, noted by pair formation, was recorded to determine females' preference for dominant or subordinate males. Females (N = 40) in the next experiment were exposed to one male experienced in ‘fatherhood’ and one inexperienced male and left to choose between them.

### Masking Experiments Using ‘Conditioned Water’

Female choice between two inexperienced males was investigated before and subsequent to a period of separation. A female was placed in a crystallizing dish (80 mm in diameter) with two inexperienced males and the male selected by the female (observed through pair formation) was noted. The three individuals were then separated and placed into crystallizing dishes containing seawater. After a period of fifteen minutes, the three individuals were returned to a crystallizing dish and female choice was again noted (N = 20). To examine the role of chemical signalling in male parental experience, the above protocol was repeated but following the initial female choice for a male, the female and the chosen male were placed in separate crystallizing dishes containing seawater. The rejected male was placed in another crystallizing dish containing ‘conditioned water’ taken from a male that was undertaking egg care. Again, after a period of fifteen minutes, the three individuals were returned to a crystallizing dish and female choice was noted (N = 20).

### Statistical Analysis

Data was analysed using SPSS v.14.0. All data was tested for normality (Kolmogorov-Smirnov test). Friedman tests were used to assess differences in aggression levels (experiment series 1) with Wilcoxon-Signed Ranks tests used to determine where the differences occurred. Frequency data from the mate choice bioassays (experiment series 2) were analysed using Chi Squared tests.
